# On the Observation of Nature in the Treatment of Disease

**Published:** 1846-04

**Authors:** Andrew Combe

**Affiliations:** Fellow of the Royal College of Physicians of Edinburgh, one of the Physicians in Ordinary, in Scotland, to the Queen, &c. &c.


### II. ON THE OBSERVATION OF NATURE IN THE TREATMENT OF DISEASE.

By Andrew Combe, M.D.,

Fellow of the Royal College of Physicians of Edinburgh, one of the Physicians in Ordinary, in Scotland, to the Queen, &c. &c.

IN A LETTER TO THE EDITOR.

Edinburgh, 25th January, 1846.

My dear Sir,—I have now carefully reperused your article in the recent Number of the 'British and Foreign Medical Review,' and rejoice that you have spoken out openly and honestly what you believe to be truth, regarding both homœopathy and what is antithetically but incorrectly called allopathy; and as I consider a consciousness of our faults to be the first step to improvement, in medicine as well as in morals, I feel no regret that you have made the confession so complete. In all probability you will be attacked for having spoken too disparagingly of "allopathy," and too favorably of homœopathy; but the result of discussion will be to extend the consciousness of such defects as are real, and to prompt to their removal, so that truth and the interests of humanity will gain by the course you have pursued. If I were to judge merely from the general tone of your remarks, I should say that you *have* exaggerated the defects of ordinary medicine, and that this has arisen from your not having been sufficiently careful to distinguish between its essence, and what may be more justly termed its errors and imperfections. Writing, as you did, for the express purpose of directing attention to the defects of ordinary medical practice, with a view to future improvement, you were in a manner forced to give them a prominence which was calculated to throw the real solidity of its foundation and the best part of its superstructure into the shade, in the minds of those readers at least who had not previously thought much on the subject, and hence were unlikely to discriminate for themselves. But, disregarding minor inaccuracies of expression and of opinion, and looking to essentials only, I should say that although, from the above causes, it might be easy to quote detached sentences from your review, which betoken an utter want of faith in medical art, yet it is evident that such was not your meaning, and that you have perhaps a stronger sense of its truth, beneficence, and importance to mankind, than many of those who will blame you for your broad avowal of its faults.

If you had been careful to make the necessary distinction between medicine as a practical science, based on the laws of nature, and only requiring the steadier application of sound principle to its cultivation, to lead to more certain and beneficial results, and that *soi-disant* medicine practised by so many of its votaries without regard to principles of any kind, and consequently often involving in its train no small amount of mischievous as well as merely negative consequences—between medicine, in short, and the abuse of medicine—and restricted your condemnation to the latter alone, you would have given less room for misapprehension and difference of opinion, and at the same time rendered your article both more acceptable and more instructive. Even as it is, however, you have not left yourself altogether defenceless. In two or three passages you admit, cursorily indeed but distinctly enough, that the defects you complain of are not inherent in, and inseparable from, medicine itself, but are only attributable to a large portion of the prevailing practice; and while you denounce unsparingly the faults and omissions of its disciples, you are so far from being sceptical of medicine itself that you pronounce it to be, even in its present state, "a noble and glorious profession," and destined to become as truly "grand and glorious in actual performance, *as it is now in its essence, its aims, and its aspirations*."

Believing your censure, then, to be directed against the faults of medical science as at present practised, and not against its principles and truths, I am so far from thinking it undeserved that I have been for years impressed with similar convictions to your own regarding them, and felt an earnest desire to contribute any little aid I could towards their correction and removal. From an early period of my professional life, I was struck with the exclusiveness with which relief was generally sought from drugs or active treatment, and the indifference with which the *lædentia* were often allowed to remain in undisturbed operation, and the *juvantia* left entirely to chance or the whim of the moment. And yet experience demonstrates that, in the great majority of cases, the drug is only one influence among many, and that it is by the intelligent regulation of these external conditions, far more than by active medication, that the physician can effectually contribute to the comfort and recovery of the patient. Disease is a perverted state of a natural organic action, and not a something thrown into the system by accident, and which obeys no fixed laws. In the cure of disease, therefore, the business of the physician is not to supersede Nature, but carefully to observe what is wrong, and to aid the efforts made by her to re-establish regularity and order. Accordingly, experience shows that the physician and the remedy are useful only when they act in accordance with the laws of the constitution and the intentions of Nature; and hence, in chronic and even in acute diseases, the most effective part of the treatment is generally the hygienic, or that which consists in placing all the organs under the most favorable circumstances for the adequate exercise of their respective functions. If this be done systematically, every effort of Nature will be towards the restoration of health; and all that she demands from us in addition, is to remove impediments and facilitate her acts.

So far, however, is this from being the prevailing view of the proper sphere and duties of the physician, that even many medical men habitually act and speak as if they considered their only business to be the prescription of drugs, or some active external remedy, such as a blister or a bleeding; and in ordinary medical education, no attempt whatever is made to direct the attention of the student to the value of preservative or preventive treatment, or to those important auxiliaries to recovery from illness which it is the province of hygiène to unfold. The consequence of thus considering drugs as our only or chief resource is, that, when called to the bedside, we are apt to fix our attention exclusively upon the prominent symptoms, and allow obstacles to recovery to continue in operation or start up unsuspected, which often go far to counteract the best devised and most active treatment. This is the more to be regretted because the practitioner himself is, or ought to be, the source of one of the most powerful and beneficial hygienic influences to which a patient can be subjected. Taking a high and just view of his position, his aim ought to be, on all occasions, to procure for the family of which he is the confidential adviser the highest health of which it is capable; and had the public, on their side, a just sense of his duties, they would resort to him for advice not only during actual illness, but regarding the management of their own health, and the education and management of their children. As a general rule, however, the practitioner attends only to the individual *sick* member to whom he is called in; and, so far from taking cognizance of the causes of disease amidst which the family may be living, he, unless specially called upon, rarely thinks of laying down precautionary rules for the *future* guidance even of the one who is ill. He prescribes for him, and the present attack once over he leaves him to the mercy of accident, to sink or swim as chance may direct. Nay, if we look into the families of medical men themselves, it is rather an exception to see any rational precautionary treatment in systematic use, or any one advantage secured, which an acquaintance with hygiène would suggest as worthy of our attention. Breathing vitiated air, for example, is universally known to be one of the most active causes of scrofula, and yet I have more than once seen the scrofulous offspring of otherwise sensible and well-informed medical men sleeping three or four in a small room, with closed curtains around their beds, while an unoccupied well-aired room was close at hand, and reserved perhaps for a stranger. Again, it is by no means uncommon to see the children of medical men suffer in health from habitual and indiscriminate indulgence of the appetite, neglect of air and exercise, and over-working of the brain, without even an attempt being made to prevent the evil by the adoption of a better regimen; and if they thus, from indolent indifference or practical blindness, neglect the protection of their own flesh and blood from evils which may be guarded against, how can we expect them to feel any interest, or use any foresight, in protecting the children of others? It is not indifference, however, that causes this inattention. It is simply that *they have never been taught* that such concern is a duty incumbent on professional men; and they have never been so taught because hygiène has ignorantly been considered to be a subject which concerned nobody but old women and hypochondriacs, instead of being, as it is in reality, both in its preservative and in its therapeutical applications, one of the most important and most beneficial elements of our professional knowledge.

It was a deep sense of the evils resulting from this state of things which led me, about sixteen years ago, to begin the preparation of the first of the three works I have since published for the purpose of inculcating the importance of physiological and hygienic information both to the public and to the profession. It was the same deep conviction which induced me in 1838, in a letter on medical education to our friend Sir James Clark which was printed but not published, to comment strongly upon some of these evils and their origin in the defective state of professional education. And it was under the same feeling that, in January 1842, when disabled from practice by the illness under which I still labour, I became so desirous to rouse attention to the subject that I embodied an outline of my views in a letter to my brother, Mr. George Combe, then resident in Germany, to be made use of by him, if I did not survive to bring them out in a more satisfactory form. This, I have never yet been able to do, although I have never lost sight of the subject; and, in this state of matters, perhaps the best answer I can now make to your appeal to your brethren, for suggestions in aid of your own efforts, is to lay that letter before you. To yourself, its perusal will be gratifying, as exhibiting the pre-existence of views in many respects in harmony with your own, as to the present state and future prospects of medicine; while to your readers, also, it may not be without interest as affording in so far a presumption of the truth of the principles which have led us both, by almost parallel although different roads, to nearly the same conclusions concerning the sources from which the improvement of medical practice is to be obtained. But to bring out this coincidence more clearly, it will be useful, before subjoining the letter, to notice briefly the analogy which subsists between its objects and the results at which you have yourself arrived.

The aim I had chiefly in view in writing that letter was to show that, before undertaking the treatment of disease, we ought to make ourselves acquainted, 1st, with the laws which regulate the action of the different bodily organs during health; and 2dly, with the *natural history or course of diseases*, that we may be able to read aright the indications of Nature in their treatment, and take special care neither to counteract her efforts nor to substitute another method of cure for hers, unless where we have positive evidence that the *vis medicatrix*, judiciously aided by us, will prove unavailing. I then proceeded to show that it is from losing sight of the order and indications of Nature, and neglecting the aid to be obtained from their observance, that no small portion of ordinary medical practice is fallacious, and some of it even hurtful, from actually interposing obstacles to the operation of the restorative power inherent in the living organism. In other words, I wished to direct attention in a special degree to the propositions, that *Nature is the active agent in the cure of disease*, as well as in carrying on the ordinary operations of life; that the physician can never be so well employed as when he acts intelligently and consistently as the *Naturæ minister et interpres*; and that there is small wisdom and less glory to be obtained from attempting either to substitute other devices for her arrangements, or to put her to the rout by main force, and by the use of means at variance with her laws.

*Your* propositions, on the other hand, are:—"1st. That in a large proportion of cases, as at present treated, the disease is cured by nature and not by the physician. 2d. That in a lesser, but still not a small, proportion the disease is cured by nature in spite of him; in other words, his interference opposing instead of assisting the cure. And, lastly, that, consequently, in a considerable proportion of diseases it would fare as well, or better, with patients in the actual condition of the medical art, as more generally practised, if all remedies, at least all active remedies and more especially drugs, were abandoned."

Such are the inferences which you have deduced from a general survey of medical practice, and I need hardly stop to point out how completely they harmonise with the conclusions at which I had arrived. This will be apparent to every reflecting reader, and will become still more so after perusing the letter in which my views are more fully developed. Here, however, it will be useful to remark that an omission occurs in your article which will tend to the further diffusion of a misapprehension already too prevalent on a point of vital importance. When you speak of "leaving the patient to the efforts of nature," as the alternative of abandoning what is usually called "active medical treatment," you, in common with most other writers, unguardedly use the phrase as if "trusting to nature" were equivalent to consigning the patient to chance or caprice for his guidance, to do anything or nothing exactly as the feeling or whim of the moment may suggest. This, is at once an erroneous and a pernicious interpretation, because it is calculated to lead to indifference and carelessness, under the very circumstances in which vigilance and discriminating attention may prove most useful. So far from sanctioning inactivity on our part, an intelligent reliance on nature implies that we shall exercise, throughout the whole course of the disease, the most watchful observation over its phenomena and progress, and not only timeously remove obstacles which may interfere with its proper course, but rigidly fulfil all the conditions which a sound physiology shows to be most conducive to the well-being of the various bodily organs, and to their restoration when disordered. In this way the physician may often exercise the most salutary influence, nay, even be the means of saving the patient's life, and yet not give one particle of medicine. In No. 14 of your *excogitanda*, and in a few other places, all this is virtually implied; but you nowhere bring out the principle with that clear and prominent distinctness which it so eminently deserves, and which is so essential to a right understanding of the question in dispute between homœopathy and ordinary medicine. I regret this omission, because the more I see, the more I am convinced that it is only by the intelligent observation and study of nature that a sound system of medical doctrine can be obtained, or medical practice ever be made to confer those benefits on mankind which it seems to me fully capable of rendering. In proportion as we proceed upon this solid basis, every step taken will be in advance, and every new discovery in physiology, anatomy, chemistry, &c., will tend to enlarge our power over disease, by enabling us more and more to give Nature fair play. Instead, therefore, of medicine being superseded, as many suppose, by taking Nature for our guide, it will, on the contrary, only begin to take just rank as a science, when our allegiance to Nature shall become practical, enlightened, and complete. One word more of preface. In reading the subjoined letter you ought to keep in mind that it was written merely as a private record of my opinions, and consequently bears the marks of haste both in its arrangement and language. If it had been intended for the public eye, I should perhaps have modified some parts of it, and been more careful in others to guard against any misapprehension which might arise from the imperfect development of my views. But as these are blemishes which do not affect the accuracy of its general statements or reasoning, and which I may safely leave to the indulgence of the reader, and as it is desirable to show the pre-existence of my opinions, such as they are, I shall not venture to make any alterations by way of removing the defects.

*" To George Combe, Esq., Mannheim.*

" My dear George, Edinburgh, 8th January, 1842.

"The great defect in the study of medicine, and in all investigations connected with it, at present, seems to me to consist in the nearly total absence of guiding principles, and in the neglect of the great rules of Bacon, and more especially of *the observation of Nature*, as the only solid foundation on which medicine or any other science can rest and advance towards perfection. This last will seem to many of my brethren a very singular charge, because if there is one circumstance on which the profession prides itself more than another at this moment, it is on the ardour with which observation is pursued and facts are sought for. Nevertheless, I believe the charge to be supported by incontrovertible evidence, and I attribute the small progress really made to this very truth. I admit that, everywhere, observations are made and facts stored up with an industry, accuracy, and zeal, which, under better guidance, would soon accomplish great things. But these observations and facts are incomplete, and therefore partial, and, if relied upon, apt to mislead. They are *phenomena* or *occurrences* rather than ultimate facts, and, their conditions and relations remaining unknown and unconsidered, they lead to no useful results. Hence, the multitude of observations daily recorded in the writings of medical men serve more to oppress the memory and puzzle the inquiring mind than to advance science and improve practice. Hence, too, the thousand and one facts of the one year disappear under the shade of the thousand and one newer facts of the succeeding year. *All*, indeed, are not of this description. Some few out of the many are *complete* facts, and have a meaning which becomes daily brighter, and bears a direct relation to practice. The sum of these constitutes the real amount of the progress made by medical science, and the proverbial uncertainty of medicine affords a pretty accurate indication of the relative amount of incomplete or false facts gathered into the granary.

"The grand object of medicine is to preserve and restore the healthy action of all the different organs and functions of the human body, so as to ensure their efficiency, and fit the individual for the successful discharge of the duties devolving upon him as a created being and a member of society. Here, then, the first step to be taken is obviously to become acquainted with the mechanism of the body, the structure of its constituent organs, the conditions or laws under which these act, the purposes which they respectively serve in the animal economy, and the relations in which they stand to each other, and to the external agents by which man is surrounded and acted upon from the moment of conception down to his latest breath. In other words, the first step towards rational principles of cure must consist in *a knowledge of the laws of the healthy functions.* The second ought to be the observation of the manner in which the various disturbing causes act upon the different functions, and the *kind, course,* duration, and termination, of the morbid action which they produce. Having investigated these points we become qualified to inquire, in the next place, what circumstances will best favour the intentions of Nature, and remove the obstacles which may have arisen to impede or thwart her efforts. To succeed in these aims, or even to make a rational attempt at succeeding, we must be profoundly impressed, or I may say *saturated,* with the great principle or truth, that all the operations and actions of the living body, whether healthy or morbid, take place according to *fixed and discoverable laws,* and that God has left nothing to chance. With this grand fact before us, it becomes palpably evident that we can do nothing rational, in the way of either prevention or cure, except in so far as we act in accordance with these laws. Many medical men have, however, a very different impression from this. A good physician will always seek to be, and never aim at being more than, Bacon's '*servant and interpreter of Nature.*' A greater than he created man and ordained the laws of his being, and no surer road can be found than that traced by the hand of his Creator. Overlooking this truth, and viewing disease as an entity ungoverned by any definite laws, and not destined to run through any definite course, many medical men talk as familiarly of *their* 'curing' and 'arresting disease' as if they had an absolute control over the whole animal functions, and could alter their laws of action at pleasure. To my mind, no clearer proof of presumption and philosophic ignorance can be found than this usurpation of the prerogatives of Deity ; and its results are often very unsatisfactory.

"That there are forms of disease in which a determinate nature and course cannot be easily traced is quite true ; but there *are* many more in which the natural course is as obvious as that of the sun. Take the familiar example of cowpox, smallpox, fever, or ague. The disease is regulated by fixed laws in such a palpable manner, that every medical book describes with perfect accuracy the appearances which each will present on given days of its progress in an average constitution. The same holds with measles, scarlatina, and many other acute affections ; and less clearly, but still perceptibly enough, with gout, rheumatism, and inflammation. All of these go through a regular course, in a shorter or longer time ; and when everything goes according to rule, we feel assured that the constitution is safer than where some unusual accident has interrupted the natural progress of events. This, be it observed, is the course towards health which the Creator in constituting man considered best for him ; and the wisest thing we can do is to act in accordance with it, and seek only to remove impediments. It is not we to whom the cure is intrusted, or by whom it is effected. The Creator has perfected all the arrangements for that purpose, and our sole business ought to be to give these arrangements full play. Man, however, is too full of his own importance to view things in this light. He wishes to be master, and to control disease by his own act, and accordingly he has in all ages been seeking for the means of 'arresting' disease at its onset. Not many years ago the cold affusion was in this way in high vogue for *cutting short* fever, and its praises were loudly sounded. Gregory applied it even in scarlet fever, and I rather think in measles. In many cases Nature was so far vanquished, by repeated cold drenching, that the disease was apparently cut short during the commotion, and, (probably from relief being obtained through another channel,) without visible bad effects. But in many more, Nature stood firm, and additional mischief was added to the original evil, in the shape of affections of internal organs, which ended fatally. Now affusion is laid aside, and its legitimate substitute, tepid or cool sponging of the surface, is usefully employed, because in harmony with the natural course of events.

"Here, then, is a type or standard to guide us to the correct investigation of nature in other less determinate affections. Is it not presumable that they also have a certain nature, and course, and termination, which it would be well for us to observe and promote? Take even a severe cold, with which all are acquainted more or less. Everybody knows that when once set in, treat it how you like, it will run through a determinate course of increase, maturity, and decline, and that all we can do is to shorten a little the duration of its stages by diminishing its intensity; or lengthen it by increasing its severity. Occasionally, it is true, an incipient cold may be stopped by a 'heroic' remedy, such as a tumbler of warm punch at bed-time; but much more frequently the heroics leave the patient worse than they found him, and the common experience of mankind shrinks from their use. Even a common boil on the fingers runs through its regular stages of inflammation and decline, or of suppuration and ulceration, each stage being hastened or retarded by external or constitutional causes, but never inverted. But if we apply to the one stage the means which are adapted only to the succeeding one, the result will be injurious; or if we lower the system so much that it becomes inadequate to carry on the regular succession of actions required for recovery, mischief must once more be produced. Let us take a case of pleurisy in an individual of average strength as an example. We know that, in ordinary circumstances, the excitement goes on increasing during a period varying from two to five or six days; that effusion of fluid into the cavity of the pleura ensues, that the inflammation then begins to abate, and after a few days more passes into an inactive state; that the natural action of the part then begins to be restored, and the fluid to be absorbed, till by and by recovery is completed. Or, if the inflammation endangers life, it either goes on longer than usual, or gives rise to effusion of a quality and quantity incompatible with recovery, and death at last ensues. In a case of average severity in a healthy constitution, left simply to the quiet and abstinence which Nature almost compels, we know, from observation, that such are the stages by which recovery is brought about; and all that the physician need attempt or care for is to use every precaution to prevent excitement from running too high or going on too long, and to meet any contingencies which may interfere and impede recovery.

"Very different, however is the general course of proceeding. Relying on the testimony of an incomplete fact (viz., that bloodletting produces excellent effects in inflammation, without attending sufficiently to the influence of the *adjuvantia*), the moment the practitioner ascertains the existence of 'inflammation,' he pulls out his lancet and bleeds the patient copiously. The oppressed vessels being thus partially emptied, much relief is experienced, and both patient and physician are pleased with the hope that the disease will be 'cut short.' This we shall suppose to have happened at the end of twenty-four or forty-eight hours or first third of the *ascending* stage of the inflammation. In a few hours, however, the vessels have contracted, and they and the heart adapted themselves to their diminished contents, and Nature thereupon resumes her attempt to carry the disease through its proper stages. The pain returns, the pulse rises, and the oppression augments. Bleeding is again resorted to with immediate relief, and the same phenomena recur. At the third bleeding we arrive at the period of the *natural* decline of the disease, and consequently no more excitement appears. 'Now then we have cut it short at last,' says the doctor, smiling complacently. 'Yes,' says the gratified patient, 'that last bleeding did the business; but what a pity you did not take more at first, and stop it at once.' With care and good management all goes on well, and by degrees the patient returns to his former diet and habits. If, however, he happens to be a person not of a robust constitution, matters go on more doubtfully, and after partial recovery he finds his strength permanently shaken, or perhaps falls into chronic disease, and ultimately dies, or after a long struggle he may regain his former health.

"If we attend to the observation of Nature with a view *to co-operate with her*, and not simply to take her by storm, we shall be guided in our practice by the indications which she presents. In smallpox, for example, or measles, the excitement often runs very high in the first or eruptive stage, and means are required to moderate it. But if we bleed too freely, it is well known that the eruption (which we shall suppose to have come out) will generally disappear and increased danger to life ensue, because the order of Nature being forcibly interrupted, some internal disease is brought on, or the system sinks exhausted. Whereas if, instead of bleeding excessively, we keep the patient very quiet, in a cool well-aired room, and administer cooling drinks, mild laxatives or antimonials, and reserve bleeding for cases of necessity, the probabilitiy will be much in favour of recovery. To apply this to the pleurisy. Instead of being intent *on cutting it short*, the moment we ascertain its existence, we would have respect to its natural course and duration, and reserve our means to carry it safely through its regular stages. So far as my observation goes, cures would be more numerous and complete were this principle followed. If a severe bleeding disturbs fatally the progress of smallpox eruption, may it not also, when unseasonably used, injuriously influence the course of internal inflammation, and lead, for instance, to fatal oppression or effusion? We know that *de facto* it is a very rare thing to cut short a smart inflammation by a severe bleeding; but is that a reason for bleeding again whenever the pain returns? I think not. If the inflammation threatens to run very high and endanger life, then depletion becomes a reasonable alternative, but not so if it resumes merely its normal or regular course. If regard were had to the nature of the disease, I am convinced that many cases in which blood is very freely drawn would do well and better by much milder means; and that in many where bloodletting is really needed, a great deal might be spared in point of quantity, with much future advantage to the constitution. Take, again, the beginning of a severe cold or influenza. We know that *de facto* it will increase for several days, after which the feverish state will decline. If, to force it away, we begin with hot drinks and sudorifics, we invariably increase the fever by adding to the excitement; but if we keep quiet in bed, moderately cool, eat little, and drink mild tepid fluids when inclined for them, abstain from mental excitement and annoyance, the feverish state will be kept down,—and if we *then* give a good sedative antimonial, we shall rarely fail to elicit free and relieving perspiration, and to break the force of the disease. If we go out during the *rising* stage, we make the cold more severe, but in the second stage we do good. In the former, meat is injurious, in the latter useful. The principle holds with a slighter cold, but is of course modified in its operation.

"Even the causes of disease, and remedies, themselves produce their effects in a regular order, according to fixed laws which we ought to study and respect; but in practice we disregard this. It is no answer to say, 'Oh, they have acted quite differently in A. B. from what they did in B. C., and therefore they can have no determinate effects.' But find out the disturbing cause which interrupted the regular course in B. C., and the uniformity will become apparent enough. Such a cause must exist whether we know it or not. Having practically no regard to the laws of the constitution, we are apt to overlook their influence on the operation of causes of disease, and to consider nothing in the light of a cause which has happened more than a few hours before. We forget that there is always an interval of hidden action in the system itself before the effect becomes palpable to sense. Take hydrophobia as a very striking example in proof. A person is slightly bitten on the finger, wipes it with his handkerchief and forgets all about it, till at the distance of two or three months symptoms of hydrophobia suddenly show themselves, and he is speedily carried off. In the interval he was unconscious of any change going on, and none was remarked by his friends. Yet nobody can deny that during all that time the poison was in the system, and producing changes of which the fatal paroxysm was the result. Even in the ordinary contagious diseases of smallpox, scarlatina, fever, &c., *several days of silent internal working* always intervene before the poison takes visible effect. This indicates the operation of fixed and determinate laws presiding over the production and course of diseased as well as of healthy actions. We never see a disease start up instantly after the application of its cause, nor full health suddenly take the place of active disease. We see the inhalation of fixed air induce immediate death; but it acts not by giving rise to disease, properly speaking, but by withdrawing one of the essential conditions of life. It is no proof of the absence of fixed laws and a determinate course to say that the same cause does not always produce the same results. The constitutional state upon which the cause acts is often different, and hence an impossiblity of the result being identical under every variety of circumstances.

"By thus insisting on the necessity of a more complete and faithful observation of the course of Nature, and of acting more systematically according to her guidance, I am far from meaning that we are to sit with our hands across, and allow things to take their own way. So far from it, it is certain that the principle I inculcate would demand more watchfulness, and give room for a nicer exercise of judgment, and a more consistent and, I believe, successful treatment. Disease arises either from the habits of the individual, from accidental causes, or from peculiarities of constitution acted upon by these. Hence, on being called to a patient, the first step in the *natural* investigation is to examine the constitutional qualities, to make ourselves acquainted with the mode of life, feelings, &c., and to trace the manner in which the cause has acted or continues to act. All these influence very greatly both the nature of the disease and its probable course. They also bear directly upon the kind of treatment and its probable success. If, however, we are content to regard disease as an entity, arising by chance and observing no laws, we shall have no inducement to trouble ourselves or the patient with any of these inquiries. Such is in fact the practical faith of the great majority of professional men. They discover the existence of an entity, which in medical works has a certain name, and, knowing that in the same books certain remedies are said to be good for that entity, they prescribe them accordingly, without giving themselves much concern about their mode of action or fitness for the individual constitution, age, or stage of the disease, and without inquiring whether there is anything in the mode of life tending to reproduce the malady or not. In many chronic ailments, removable causes are thus often left in full operation, while the effect is partially mitigated, but not cured, by the use of active medicines, and in a short time the whole evil returns in its full force.

"Whereas, if, proceeding according to the order of nature, we can trace the disease to any error in the mode of life, to any external source of danger, or internal peculiarity of constitution, aggravated by either of these two conditions, we can convince the patient of the fact, and give him a rational and confiding interest in the changes which we may recommend, and thus not only promote his recovery, but render him proof against all the seductions of quackery. According to the prevailing kind of intercourse between patient and physician, viz., unhesitating dictation on the one hand, and ignorant obedience on the other—blind faith is the pivot on which their mutual connexion turns, a faith which is thus necessarily at the mercy of the chapter of accidents, and is often supplanted by reliance on the first bold and confident quack who comes in the way. People wonder that quackery abounds, and medical men ask for power from the legislature to put it down. They themselves, however, are in no small degree its abettors, and they have the remedy already to a great extent, although not wholly, in their own hands. *If they, who are educated, and should know better, accustom their patients to the principles of quackery, by themselves treating them empirically,* can they wonder that patients who are not professionally educated, and are trained and treated on purely empirical principles, should be as ready to listen to the assurances of the quack as to those of the regular practitioner, whose manner of proceeding is often so nearly allied in kind, as to present no very obvious marks of distinction from that of the quack? In fact, medicine, as often practised by men of undoubted respectability, is made so much of a mystery, and is so nearly allied to, if not identified with, quackery, that it would puzzle many a rational on-looker to tell which is the one and which the other. And this being the case, it requires no ghost from another world to explain why the profession has decidedly sunk in public estimation, and does not exercise that wholesome influence on public opinion which it ought to do. If the mass acts empirically, it can, in the very nature of things, expect only the amount of respect due to empiricism. The public mind has advanced immensely within the last fifty years, in elevation of view as well as in extent of knowledge. Medicine, however, has advanced only in knowledge; and, on looking back to the writers of 80 or 100 years ago, I incline to think that it has actually *lost* in elevation and comprehensiveness, and even in the perception of its own *nobleness* of sphere. If this be so, we must look within for the sources of regeneration, and for the means of regaining a dignified and honorable place in society. The public mind has advanced, while, in scope and general principles, the professional mind has stood still. To regain respect and relative position, the latter must shoot a-head again, and, on doing so, will regain its influence also.

"Let us, however, return to the case of pleurisy as an illustration. It is in general a well-marked disease; its nature is supposed to be well known, and the indications of treatment as clearly understood as those of any malady to which the human frame is liable. It is, therefore, rather a favorable example of the state of professional knowledge and principles of treatment. And yet, what do we find? Are medical men agreed how it should be treated? They *ought* to be, as it is frequent enough in its occurrence to give ample opportunities for experience; but they are not. In this country many place their chief reliance on free and repeated bloodletting and mercury. In France the plan of bleeding *coup sur coup,* in small quantities frequently repeated, is somewhat in vogue. In Italy bleeding was given up by many, and large doses of tartar emetic were resorted to. In Germany the cure was often intrusted to homœopathic doses of 'medicaments.' The strange thing is, that pleurisy is cured by, or at least pleuritic patients recover under, each of these plans, while also many recover under the 'médecine expectante' plan of lying in bed, drinking ptisan, and waiting upon Providence. Even in this country, however, a change has come o'er the spirit of my brethren within my own brief day. When I first opened my professional eyes, the lancet was in great vigour, and a well-employed medical man almost lived in a stream of blood. '*Vigorous practice*' was the order of the day. In typhus as well as in inflammation the lancet was the sheet-anchor of many, and quantities of strong purgatives were administered, sufficient to put disease of every shape and hue to the rout. Take the same men of vigour now, at the distance of twenty-four years, and they will tell a different tale. It is no longer, 'Be bold and decided and prompt in what you do;' but, 'Be watchful and trust *something* to nature.' This diversity of opinion and practice seems to me to have arisen partly from different constitutional states, arising from changes of atmospheric or other influences affecting the prevailing character of diseases, but much more from all parties disregarding Nature's indications and efforts, and acting heterogeneously and without *any* rational principle. In this way, I believe that under each plan of treatment individual cases recovered which would have terminated fatally under a different mode; but also, that under all of them many died who might have been saved by a more rational and close adherence to sound physiological principles. To these we may be partially led even by a reference to the symptoms usually present. The sense of cold and shivering, which commonly precede, would lead to the avoidance of exposure. The pain, increased by breathing, inculcates absolute rest and refraining from speaking. The oppressed respiration requires, of course, *purity* of air more than ever. The heat and thirst which soon arise demand cooling simple drinks, and occasionally tepid sponging of the arms and face. The local stitch asks for mild emollients, such as bran poultices, and the impaired appetite requests the stomach to be let alone. If the bowels are oppressed, a mild laxative or lavement are indicated.

"Suppose the exciting cause not to have been violent or long-continued, and the attack, consequently, one of moderate intensity in an average constitution; the probability is, that by the adoption of the above means alone the disease will run through its course safely, and leave the constitution unimpaired. In this case effusion of fluid will as usual take place, be moderate in quantity, and be gradually absorbed. This effusion of serum, be it observed, is a natural provision—1st, for the relief of the vessels of the inflamed part; and, 2dly, for preventing adhesion between the pleura of the ribs and that of the lungs, and, consequently, for the preservation of their free play upon each other. When two surfaces, one of them inflamed, are placed in juxtaposition, and no fluid intervenes, adhesion takes place and hampers their mutual action. Within certain limits, then, there is no advantage to be gained by *preventing* the effusion, supposing we could always do so. *If we watch attentively*, and find all going on regularly and smoothly, according to the method by which Nature removes the inflammation, why should we step in to prevent, say, any effusion? I believe that many of the extensive adhesions actually met with after death, in persons who had at one time suffered from active pleurisy, are the results of over-active treatment interfering disadvantageously with natural processes. Adhesions occur also, however, but generally of small extent, from inflammation so slight in degree as to have suppressed the usual secretion, without leading to any effusion at all.

"Again, suppose the exciting cause to be very energetic, the habit inflammatory, and the attack violent. We, of course, ought to be doubly rigid in the use of the means above mentioned, and in addition we must watch closely, and come in time to Nature's assistance to prevent mischief. The reaction may run so high as to induce disorganization, or the effusion of an albuminous or semipurulent fluid, or to over-excite the brain or heart, and produce distinct disease in them. Or the individual may have some infirmity of body, which renders it more dangerous to wait the usual course of events than to attempt to shorten or arrest it. The thinking physician will take all these contingencies into account, and decide to act; but when he does so, he will still time his measures according to the law of Nature.

"18*th January*.—So far I had got, bit by bit, in my exposition; but as writing fatigues me I must now break it short, and send this away. Keep it merely as a rough and unfinished outline, and not as a proper embodiment of my views. To bring out these would carry me into writing not a letter but a book. I shall, therefore, not resume the subject, nor for a time must you expect any more letters from me.

"Yours, &c. A. C."

Assuming the general accuracy of the views contained in the preceding letter, we shall be warranted in inferring from them that the radical fault of a large portion of the prevailing medical practice consists—1st, in prescribing for the abstract disease, irrespective of the constitutional condition of the patient; 2dly, in placing the chief reliance, even in cases where the active interference of the physician is not at all required, on the use of drugs and active means, the operation of which, from their administration being guided by no principle or well understood rule derived from experience, tends not unfrequently to impede and disturb the restorative efforts of Nature; and, lastly, in the general and often entire neglect of those physiological or hygienic arrangements which, from their co-operation with the intentions of Nature, contribute so powerfully to the well-being of the individual when he is in health, and to his recovery when attacked by disease. But much as these defects impede the present efficiency of medicine as a practical art, they do not in the least disprove its firm foundation in the laws of nature, or detract from the value of its principles, when properly applied (as they also often are) under the guidance of reason and experience. On the contrary, by laying bare the causes of the blemishes by which the medical superstructure has been disfigured, we not only show how they may be removed, but hold out the prospect of medicine being ultimately brought to as high a degree of certainty and perfection as any *estimative* science can attain. For even were all its constituent elements as fixed and certain as those of mathematics, their application must still be made by men of varying powers and knowledge to patients of different ages, sexes, habits, and constitutions; and it is plain that, under circumstances so inherently variable, there must always be such a wide field for the exercise of individual judgment and skill, as to render absolute similarity or equality of practice a moral impossibility. Approximate results are all that can be looked for.

Keeping to essentials then, and believing as I do, from the force of evidence, that ordinary medical science rests on a solid and natural foundation, I see great reason for endeavouring to improve and purify its doctrines and practice, but none whatever for abandoning the principles on which it is based. So far from this, I am more than ever persuaded that the observation of the order of nature is at once the surest guide we can have in the study and treatment of disease, and the only means by which we can arrive at a correct estimate of the special action and relative value of every therapeutic agent, whether homœopathic or eclectic. The same remedy prescribed at different stages of the same disease may be followed by consequences so widely different as wholly to mislead us if we do not use the necessary discrimination. Thus an infinitesimal dose or an ordinary drug administered just at the crisis or turning point of a disease will be *followed* by an alleviation of all the symptoms; but surely it would be unreasonable to infer, merely from that sequence, that the dose or the drug was therefore *the cause* of the amendment. On the other hand, when a medicine in itself useful or harmless, given eclectically or homœopathically during the increase of the disease, fails to prevent that increase from taking place, are we on that account warranted in inferring that it was the *cause* of the subsequent aggravation of the symptoms? Certainly not; and yet such inferences are so frequently drawn on equally slender grounds, that I, in common with most of my brethren, have had the mortification to receive unmerited praise and gratitude on the one hand, and blame on the other, for good and bad consequences which I was conscious were wholly independent of and unconnected with the drugs which were supposed to have caused them. In both instances all that could be *justly* said was, that the phenomena which ensued were *subsequent in point of time* to the administration of the medicine; and hence, unless we discriminate carefully between mere sequence and actual causation, we may easily be led by supposed experience into the most grievous errors. A case illustrative of this principle occurred a few days ago. A young lady complained of troublesome palpitation of the heart, brought on by unusual agitation of mind. At the very time when the exciting cause was removed, a homœopathist was consulted and prescribed a globule. In a day or two the symptoms began to subside, and in a few days more they ceased. The homœopathist knew nothing about either the existence or cessation of the exciting cause, and therefore would naturally consider the result as produced by his medicine. In reality, however, the supposed cure was so palpably the mere return of quiescent and regular action in a healthy subject, after the exciting and disturbing cause had ceased to operate, that the homœopathist himself would never have thought of ascribing it to anything else, if he had been fully aware of all the facts of the case.

It is from not taking sufficiently into account the many modifying causes which may influence the progress and results of disease, altogether irrespective of any drugs which may have been administered, that the most opposite conclusions may be honestly arrived at regarding the efficacy and merits of the treatment pursued. In the preceding letter, taking smallpox as an example of a disease known to pursue a regular course, I contrast the safety and advantage of a treatment conducted according to the indications of Nature with the mischief which may be done by interfering violently with her order of proceeding; and show how differently the same indications may be interpreted when we have no sound principle to guide us. A few weeks ago two cases of this disease occurred, so strongly illustrative of both propositions, and so entirely in harmony with the views we are both advocating, that I cannot refrain from shortly alluding to them. The first patient was attended by a general practitioner, who, it was said, had improperly resorted to copious and repeated bloodletting at the beginning of the attack. The eruption did not come out freely, and the patient sank. The second case occurred in the same family, but was attended by a judicious and watchful homœopathist, who adopted what I have described as the natural treatment, but with the addition of certain infinitesimal doses. The result was that the disease went through its regular course, and terminated in complete recovery. By some of those interested in the two patients, this result was proclaimed to be a triumph of homœopathy over "allopathy;" but, considered impartially, and assuming the accuracy of the statements made to me, the triumph, if there be any (for it does not follow that because the first patient died under the treatment pursued she would *necessarily* have recovered under the homœopathic), may more justly be regarded as that of rational over irrational practice. Not having seen either of the cases, and not being accurately acquainted with their details, I shall not pretend to pronounce a positive opinion concerning the influence of either the bleeding or the globules; but as the results in both instances coincided exactly with those specified in my letter, written four years before they occurred, as likely to attend *good* as contrasted with *bad* general treatment, I can scarcely be accused of uncharitableness in still doubting whether the globules had any real share in bringing about the recovery which report ascribed wholly to their agency.

Connected with and arising out of the same neglect of the natural history of disease is another difficulty, which greatly impairs the value of many of the cases published as illustrative of the efficacy of either system of treatment. In the majority of instances the vagueness and meagreness of detail are such as to render it impossible for the reader to form, *from the data before him*, even a rational conjecture how the disease has arisen, or what constitutional or external causes are in operation to influence its intensity and affect its duration. It is precisely the obscurity inseparable from these omissions which often renders it difficult for the practitioner to appreciate the real agency of a new remedy or mode of treatment, where the unprofessional friends, from unconsciousness of their own ignorance, see only the clearest evidence of its efficacy, and are apt to express amazement and indignation at what they consider the wilful blindness or prejudice of the practitioner who, still feeling a doubt, asks for further evidence before sharing in their enthusiasm. Not aware of the many sources of fallacy which lie in the way, the bystander attaches unhesitating faith to the narratives of cure, the very marvellousness of which is sufficient to call for the exercise of a prudent caution before perilling the lives of others on the assumption that the narratives contain a pure embodiment of truth.

It is for this reason, also, that I consider the numerous cases published in some recent homoeopathic works to be individually valueless as *proofs* either of the action of homoeopathic remedies, or of the superiority of the new system to the old. Taken in the aggregate, their results may constitute a fair ground for further inquiry; but, individually, little weight can be attached to them as proving the specific virtues of any particular medicine, or the reality of its influence on the cure. They contain a mere statement that certain symptoms were complained of, and certain remedies prescribed, and that certain changes followed; but they afford no concomitant information regarding the mode of life and causes which led to the disease, or the hygienic observances by which the exhibition of the remedies was accompanied; and, consequently, the reader is not enabled to form an opinion of his own as to the agency by which the cure was effected. To the homoeopathist himself, who watched the cases, they may be pregnant with meaning, because he may have satisfied himself on these points; but to his readers they are nearly barren as items of evidence. This, indeed, is a defect inherent in a large proportion of ordinary as well as homoeopathic cases, although in a smaller degree; and as a consequence cures are often ascribed to the use of drugs, which have in reality had nothing to do with them. A case in point occurred to myself shortly before I relinquished practice. A delicately-constituted boy fell into a state of health which excited the alarm of his friends, who consulted several practitioners about him without benefit. The treatment prescribed seemed to have been in many respects appropriate, but little advantage was derived from it. This led me to minuter inquiry, and I found that the guardians of the boy, in their anxiety for his mental progress, kept him at school from an early morning hour till late in the afternoon, and thus prevented him from obtaining a due supply of nourishment to support his strength, till, by the mere lapse of time, he became exhausted and irritable. They conceived a biscuit or piece of bread in the forenoon sufficient for him, because it seemed to be so for other boys. I prescribed a mild tonic for immediate relief, but insisted that he should have a longer interval in the forenoon for exercise in the open air, and an early dinner of plain nourishing food. The decided amendment which speedily ensued was attributed by some of the friends *to the medicine;* but, in reality, it was due almost exclusively to the regimen being brought into harmony with the laws of nature. Similar medicines prescribed by others had been of no use; but, conjoined with the required change of regimen, their operation, so far as it went, was beneficial. Every medical man must have met with similar cases, and also with many in which it remained difficult for him, even after the most careful consideration, to determine what was the real agent in the recovery which ensued. If, in the case of the boy, I had prescribed a homoeopathic globule instead of the simple tonic, recovery would, I believe, have equally followed: but would the infinitesimal dose have been on that account entitled to the credit? I refer to this because I am acquainted with several cases of homoeopathic cures equally equivocal as this would have been, but to which much importance has nevertheless been attached; but I need not occupy your space with their details. To my mind they do not indeed *disprove* homoeopathy, but they do show that Nature, duly seconded by the arrangements of the practitioner, is adequate to the cure of many diseases, without his resorting to drugs of any kind.

Speaking, then, from a general view of medical practice, I should say that it is open to the charge of being carried on without due regard to the period and natural course of the disease, or to any other recognized principle which can yield us safe guidance, and that this is the cause of much of the uncertainty and contradiction for which our art is proverbial. We too often, I repeat, attack the disease as if we had to deal with an entity, and not with a state *of a living being* of a determinate constitution, who is suffering under it, and whose qualities, tendencies, and powers of endurance, consequently require to be taken into account as well as the disease itself. These defects, however, are not inherent in and inseparable from medicine. They are simply the defects of its cultivators; and if we were to begin by making ourselves acquainted with the laws of action of the different bodily organs, with the natural history of the diseases to which they are liable, and with the physiological conditions most favorable for their restoration, and then endeavour to deduce our curative indications from a general consideration of all those circumstances, there is every probability that we should make a nearer approach to unanimity of opinion, because then every one would set out from the same starting point, and proceed in the same direction towards the goal. It is true that as yet we know so little of the course and tendencies of many forms of disease, that we might often be at a loss what treatment to adopt; but the clear recognition of our ignorance is the first step towards the acquisition of knowledge, and more enlightened observation would gradually remove the obscurity which at present prevails.

If any of these remarks should be considered by some to imply a want of faith in professional aid, I have only to reply that no conclusion can be more unfounded. With all its imperfections on its head, medicine, in the hands of discriminating and experienced men, seems to me already to be the source of the greatest benefits to suffering humanity. When cultivated with more constant reference to sound principles it will become still more beneficial in its applications. It is a deep conviction of this truth which makes me so desirous to assist in the good work of medical improvement to which you are now devoting yourself

It would be easy for me, were it needful, to point out numerous instances in practice, in which medicines were prescribed without reference to any guiding principle, or natural tendency in the system at the time, and in which, consequently, results ensued which were wholly unexpected. In this respect I cannot exempt myself from the censure I have bestowed upon other practitioners; and I must further admit that, even after I became fully alive to the importance of endeavouring on all occasions to act as the assistant and interpreter of Nature in the treatment of disease, I continued to meet with many cases in which I could not discover what the real order of Nature was, and in which, consequently, I was obliged to resort to purely empirical treatment, and with necessarily varying success. But even then I had this advantage on my side, that the abiding consciousness under which I lived of Nature's presence and power inspired me with watchfulness for the observation of her earliest indications, and induced me in the meantime to borrow all the aid I could from her, by placing the various bodily functions, as far as possible, under the conditions most favorable for their healthy operation. When doing so, I have sometimes been rewarded by the gradual disappearance of difficulties which seemed at first irremediable, and by an amount of improvement which served to increase my faith in the restorative powers of Nature, even under unfavorable circumstances.

The one great principle, then, to which a comprehensive review of homœopathy, "allopathy," hydropathy, and all other systems of medicine seems irresistibly to lead is, that in all cases and on all occasions, *Nature is truly the agent in the cure of disease; and that, as she acts in accordance with fixed and*

*invariable laws, the aim of the physician ought always to be to facilitate her efforts, by acting in harmony with, and not in opposition to, those laws.* Disease, as already remarked, is a mode of action of a living organism, and not an entity apart from it. In accordance with this view, experience shows that when we favour the return to a normal action by simply natural means, recovery will ensue in most cases, without the use of drugs at all. So far from being always necessary to a cure, drugs are required only where the power of Nature to resume her normal action proves inadequate or is impeded by a removable obstruction. Even then it is still Nature acting in accordance with her own laws that brings about the cure. She may be *aided,* but *she ought never to be thwarted;* and medicine will advance towards the certainty of other sciences only in proportion as we become saturated with this guiding principle.

A few words now on homœopathy in particular. I am very glad that you have brought the question of its truth and merits seriously before your readers; for of all methods of advancing the interests of science, that which consists in the supercilious neglect of alleged new discoveries, merely on the ground that they differ from what is already known, is assuredly the worst. We know far too little of the constitution of nature, and more especially of animated nature, to be able to decide *a priori* what can or cannot be true regarding the mode in which vital operations are conducted, or in which they may be modified by external influences. Medicine itself is in its very essence an estimative science, and the truth of the principles on which it rests can be ascertained or verified only by careful and extensive observation. Theoretically these principles may be rendered more or less probable in the eye of reason, but they never can be demonstrated except by an appeal to experience. Medicine, moreover, considered as a system or body of doctrine, is still at the best in a very defective state. Every page of your Review admits and laments this unfortunate truth. We ought, therefore, to extend the hand of welcome to every man who is able either to correct an established error, or add a new truth to the existing store; and much more so, if the offered contribution should be that of a new and important principle capable, if true, of modifying and improving the whole field of medical practice. Not that we are by any means called upon to run after and examine every new theory or alleged discovery in medicine, merely because it is announced to be such. If we did, we should impose upon ourselves a never-ending and most useless task. But surely we are bound not to be too rash in rejecting without examination facts and principles which come before us, attested by men of experience, skill, and integrity, and who can have no motive for deceiving us. Judged of by the standard of our own opinions, these facts and principles may seem at first sight to be altogether absurd; but if so, the question then comes to be, is our standard itself undoubtedly a correct one? Or may it not be that ignorance has misled us to adopt it as infallible, and that it would be wiser in us to compare both it and the alleged discoveries with nature before assuming either to be demonstrably true? Had this reasonable course been followed with the discoveries of Harvey, Jenner, and Gall, how much idle and acrimonious disputation and professional obloquy might have been avoided, and how many benefits might have been obtained which were lost for years to suffering humanity, by the opposite course of first rejecting and ridiculing, and then examining evidence only when compelled to do so by a humiliating, because tardy and ungracious, necessity.

Let not this wholesome lesson, then, be lost to us who are the living successors of those who acted so unwisely. To use your own words, homœopathy, whether true or false, comes before us for examination with "claims on our attention which cannot be gainsayed." It is, you say, an ingenious system, "professing to be based on a most formidable array of facts and expements," and "woven into a complete code of doctrine with singular dexterity and much apparent fairness." Its discoverer and chief cultivators are, as you believe, "sincere, honest, and learned men." Dr. Fleischmann, perhaps the most eminent among them, is considered by you as "a regular, well-educated physician, as capable of forming a true diagnosis as other practitioners," and as "a man of honour and respectability," whose testimony as to matters of fact you "cannot therefore refuse to admit." Even in acute diseases the results of his treatment are such as "would have been considered as satisfactory by any candid physician;" and, according to you, even his own narrative affords ample evidence that many of his cases *were* severe; and you candidly add that this was confirmed to you by the private testimony of a competent physician who followed his practice for three months, and himself traced the progress of the pneumonic cases by careful auscultation through all their stages up to perfect recovery, which took place in as short a time as under the most energetic treatment. In some eruptive and febrile diseases the mortality is stated by you to have been below the ordinary rate. Let us scout quacks and pretenders as we may, here is surely too strong a *prima facie* case to warrant our dismissing it with mere ridicule and contempt, and one which amply justifies you in the course you have adopted of seriously investigating its claims. I am aware that you have been blamed by many for occupying your pages with even a refutation of "such trash;" but so far from participating in this feeling, my chief objection to your review is that it does not go far enough to be *conclusive,* either for or against homœopathy. You have admitted too much, and denied too much, to warrant your either pronouncing a definitive sentence, or reposing in *mere opinion* against its truth. Had you shown that the general results of its practice were *less* favorable than those of ordinary medicine, you might legitimately have held yourself absolved from going further; but in your present position you can no longer stand still. If, as you admit, the truth of homœopathy is a *question of fact and experience,* which no mere argument can set aside, you are bound in reason and in logic *to test its facts* for yourself before pronouncing authoritatively that it is not true, and more especially before stigmatizing it as "useless to the sufferer and degrading to the physician." However improbable its doctrines and practice may be in an *a priori* point of view, it is not by argument or ridicule that its alleged stronghold of facts can be successfully assailed. *As a matter of theory,* supported only by argument, homœopathy produces no conviction whatever on my mind of its truth, or even of its probability; but as *a question of fact,* claiming to rest "on the irresistible ground of its superior power of curing diseases and preserving human life," and on the alleged experience of able and honest men, as competent to judge as most of those who oppose them, I cannot venture to denounce it as untrue, because I have no experience bearing especially upon it to bring forward, and we are still too ignorant to be able to predicate *a priori* what may or may not be true in the great field of nature. But after the presumptive evidence which you yourself have produced, if I were now in practice I should hold myself bound, without further delay, to test its truth by careful and extensive experiments; because where truth is really our aim, the shortest and least encumbered approach to it is always the best; and even a few well-defined and carefully observed facts would carry far more weight, as *items* of evidence, than volumes of general or controversial reasoning. In instituting such an inquiry, however, we ought to be prepared to lay aside prejudice, and to scrutinize facts with the fairness and liberality characteristic of a love of truth, and not regard them with dislike and distrust, as if they were as many live embers purposely laid down to burn our fingers the moment we touched them. View the question as we may, *one of three things must be:* either homœopathy is true, or it is false, or it is a mixture of truth and error. Let us suppose the worst, and hold it to be false in its foundation, and false in its superstructure, what harm can result from putting it to the test, and ascertaining the fact demonstrably? None whatever, but, on the contrary, much good. We shall at least *have gained the power of giving a direct and authoritative negative to its allegations,* which we shall then prove to be fallacious, and which have been suffered to reign and diffuse themselves for thirty years from the absence of *direct* counter-evidence by which to rebut them. We shall thus be able also to put the profession and the public on their guard with some chance of being listened to, and shall have obtained the inestimable advantage of keeping our own minds open to the admission of new truths, and of showing that in our estimate of evidence, and in our conclusions, we are actuated not by any mean jealousy or dogmatic assumption of authority, but by the single and simple desire of advancing the interests of science and humanity to the best of our ability. The *very worst* that can happen in the event of its being wholly untrue is, that we shall have bestowed some time and pains in obtaining the means of more effectually putting down a great error; while, as a compensating advantage of no small value, we shall have at once increased our knowledge and cultivated and strengthened our intellectual and moral faculties, by the very nature of the mental exercise which such a scrutiny requires; and surely these will be rewards well worth all the time and trouble which they may cost us.

If we adopt the supposition that homœopathy embodies an *admixture of truth and error,* the inducement to institute a rigid and careful inquiry into its claims becomes still more imperative, that we may obtain possession of the one and carefully avoid the other. The degree of success, be it more or less, which all admit to attend homœopathic practice, as conducted by such men as Fleischmann, is sufficient to show that either the system or its advocates possess *some* advantages in the treatment of disease, which it would be useful for ordinary practitioners also to examine and adopt. Whether the means which afford these advantages be derived from the domain of hygiène, of materia medica, or even of the imagination, is of comparatively little practical consequence, provided *their utility to the patient and the best mode of reproducing and applying them to the treatment of disease* can be clearly established. This, however, can be done only by careful investigation; and that such investigation would be amply rewarded may fairly be presumed, from the good already effected by homœopathy in demonstrating the evils attendant on that over-active medication, which characterizes so much especially of English practice. Ordinary medicine is now not nearly so heroic and undiscriminating in the use of strong measures as it was some years ago, and this improvement is unquestionably due in part to the progress of homœopathy, as well as to the natural increase of our knowledge.

The remaining, although unlikely, supposition, viz., that homœopathy shall prove to be *essentially true* in its fundamental principle, and consequently fraught with benefits to science and humanity, as its advocates affirm it to be, need not detain us more than a moment *If true,* how much more shall we then have reason to rejoice that we did not look upon its claims with prejudiced eyes, or reject and condemn it unheard and unexamined! Had Harvey's detractors examined his facts first, and then given their verdict, how different would the results have been to themselves, to him, and to mankind! And yet in our own day the profession acted towards Jenner, and also towards Gall, as if Harvey's name and memory had been blotted from the page of history.

I press all these considerations upon you, not from any particular leaning towards homœopathy, or any other new and disputed branch of knowledge, but because of the transcendent importance of cultivating science in a right spirit, and offering truth a ready and unprejudiced welcome from whatever quarter it may come. Ridicule and declamation may be rightfully employed to explode errors *after they shall have been proved to be so;* but they are most unfit instruments for the primary investigation of truth, and as such ought to be banished for ever from scientific discussion, and a candid spirit of philosophical inquiry be instituted in their room. I have had no personal experience of homœopathy, and am, consequently, as little inclined to admit as to reject its claims, but I should wish to steer clear of prejudices regarding it. There are perhaps a few analogies in its favour, but its doctrinal expositions embody much that is crude and contradictory, and most of its practical evidence, in the shape of published cases, is rendered nugatory by the same sources of doubt which render so much of professional experience and testimony inconclusive, if not worthless. Sufficient discrimination is not used, or if used not recorded, to warrant much reliance on the alleged connexion between the remedy and the recovery in individual cases. As in ordinary medicine the *post hoc* is too universally assumed to imply the *propter hoc.* If I am not mistaken, the more intelligent homœopathists themselves admit this, and in consequence do not claim *belief* on the ground of the recorded cases, but affirm that on the contrary rational belief can be produced only by personal and extensive experience. But while I refuse belief I can see no reason for that deadly hostility which many feel towards *the principle* of homœopathy. If it be true, such hostility is misplaced and injurious. If false, it is needless and supererogatory; for the hostility will vanish with the non-existence or destruction of its object. And, after all, why should either party delight in representing homœopathy and ordinary medicine as *in every respect* opposed to each other? In a large proportion of cases the more rational and enlightened men of both parties employ the very same hygienic and general means which we have already seen to act so large a part in effecting recovery; and the chief difference between them relates to the principle on which the requisite medicine is to be selected. The homœopathist prescribes according to the principle of *similia similibus*, because experience, he says, proves this to be the safest and most efficacious plan. The ordinary practitioner, on the other hand, prescribes that which rational, or it may be routine, experience has led him to believe the best adapted for cases of the kind before him; and without stopping to inquire whether its action is homœopathic, allopathic, or antipathic. Surely there is no necessary cause of quarrel in all this, but merely results to be tested by careful experiment. "True," you may say, "but then the infinitesimal doses are so absurd." They certainly look very absurd; and I at once admit that nothing short of demonstration and personal evidence will ever inspire me with a conviction of their power to do either good or harm. But then all homœopathists say that it is the principle of *similia similibus,* and not the dose, which constitutes the essential element in their system, and that the infinitesimals may be discarded and yet the great principle of homœopathy remain unshaken. This latter, then, is the great fact to be proved or disproved, to settle the question for ever; and why should it not be put to the test? Let experiments be made on a sufficient number of healthy persons with quinine, or any other drug, to ascertain whether it really has the property ascribed to it of exciting certain groups of symptoms in a sound constitution, and after carefully varying and repeating the experiments, faithfully record and publish the results. Surely there is nothing unphilosophical or undignified in instituting such an inquiry, and nothing so difficult as not to be easily overcome by judgment and patience. Having tried their action in health, try the same remedies *in the usual doses* in the treatment of disease with as much care and discrimination as possible, and again record the results. If the principle holds good, let us adopt it and be thankful we have now a surer guide than before. If it fails, our exposure of its fallacy will tell with tenfold effect, from being founded on direct experience. In the same way with the infinitesimal doses, let us go at once to facts, and leave mere disputation to the idle speculator. All truth is harmonious, and what is true in the one system must harmonize with and throw light upon what is true in the other, and, consequently, it would be better for science were both parties to endeavour to find out the points of contact rather than those of repulsion. In the very nature of things, certainty or absolute identity of opinion is, and ever must remain, an impossibility; and it ought never to be forgotten that in this respect there is a radical difference between medical and physical science. Physical science is *fixed* and *positive* in its principles and in its details, because its facts are always accessible for examination under the same conditions, or under such variations as can easily be traced and allowed for. Medicine, on the contrary, is and ever must be an *estimative* science, because its facts and phenomena are subject to continual variations from varying states of the body and mind of the patient, which we can neither control nor appreciate with entire accuracy. Its cultivators, too, are men differing in intellectual power, knowledge, skill, and experience; and even if they were all equal, their judgment is constantly liable to be impaired or disturbed by any slight disturbance of health or excitement of feeling, or even by a little extra fatigue, and hence, although its principles are fixed and determinate, because also founded on the laws of nature, the soundness of the conclusions deduced from them for the guidance of treatment must ever depend on the soundness of the estimate formed by the physician of their operation and influence in the individual case. Very rarely indeed can they be absolute, and hence the wide field for the exercise of sound judgment, skill, and discrimination on the part of the practitioner, and the mischief which may attend a practice founded on mere routine. Hence the forbearance and charitable construction which, as members of a liberal and useful, but most difficult profession, we are bound to exercise towards each other; and for the exercise of which there is, I fear, ample occasion in this very letter. But restrained as I have been by impaired health, as well as by the impossibility of doing full justice within your limited space to a subject at once so extensive and so important, I could not always express my opinions with the precision which I wished, and therefore I must trust to your good sense and right feeling not to give undue importance to any isolated or dubious expression which you may meet with, but to adopt that meaning which is in accordance with the general spirit of my remarks. My only anxiety has been to help you in the good work to which you have dedicated yourself with so much zeal, energy, and talent, and for which you will, I have no doubt, one day have your reward in a rich harvest of useful results.

I remain, my dear sir, very sincerely yours,

Andrew Combe.

P.S. To prevent the recurrence of a very common mistake, may I be allowed once more to call the attention of your readers to the broad distinction which subsists between the principle of *similia similibus*—which alone constitutes the basis of homœopathy—and the doctrine of the infinitesimal doses, which has been engrafted on, but does not constitute a necessary part of it? This caution is the more required, because the two propositions are more frequently confounded than distinguished, and we are surely bound to take the word in its correct meaning, as used by Hahnemann and his followers.

In a practical point of view, also, it is important to note the distinction, because, while it would be comparatively easy to verify the specific powers or mode of action of any drug given in ordinary or appreciable doses; and thus to *test the real principle* of homœopathy, it would be far more difficult and require a much longer and more varied inquiry to obtain precise and conclusive proofs, were the same drugs to be administered in doses altogether inappreciable to sense, as in the decillionth of a grain. We ought therefore to begin with the most important part of the inquiry first, and to leave the doctrine of the infinitesimal doses to be tested in its turn, if need be, after the *viability* of its parent shall be decided.

